# Giant‐cell arteritis with toothache

**DOI:** 10.1002/jgf2.684

**Published:** 2024-03-13

**Authors:** Jun Usami

**Affiliations:** ^1^ Primary Care Center/General Medicine Aichi Medical University Hospital Nagakute Japan

**Keywords:** fever of unknown origin, giant‐cell arteritis, temporal artery biopsy, toothache, ultrasonography

A 78‐year‐old woman visited the outpatient clinic with a 4‐week history of low‐grade fever of unknown origin. One week after fever onset, she visited the dental clinic because of upper left toothache and left jaw claudication, and she was scheduled to have a mouthpiece made. No dental caries was observed. Since the cause of fever was not clear, the patient visited our clinic. On presentation, she was alert, and her vital signs were normal. Physical examination revealed a slightly enlarged left temporal artery without pulsation and tenderness (Figure 1A). She did not complain of pain and morning stiffness about the shoulders, neck, hip girdle, and proximal thighs. There were no complications of polymyalgia rheumatica. Her C‐reactive protein level was 4.8 mg/dL (reference: 0–0.3 mg/dL), and erythrocyte sedimentation rate was 85 mm/h (reference: 0–10 mm/h). Ultrasonography revealed thickened walls in both the temporal artery frontal branches (Figure 1B; left temporal artery). Giant‐cell arteritis (GCA) was considered likely.^1^ Left temporal artery biopsy was performed, which revealed a highly narrowed lumen. Lymphocytes, neutrophil infiltration, and granulation tissue formation were observed in the blood vessel wall, and multinucleated giant cells were also observed. The biopsy results confirmed the diagnosis, and 40 mg prednisolone (1 mg/kg body weight) was orally administered daily.^1^ Symptoms resolved within a week.

**FIGURE 1:**
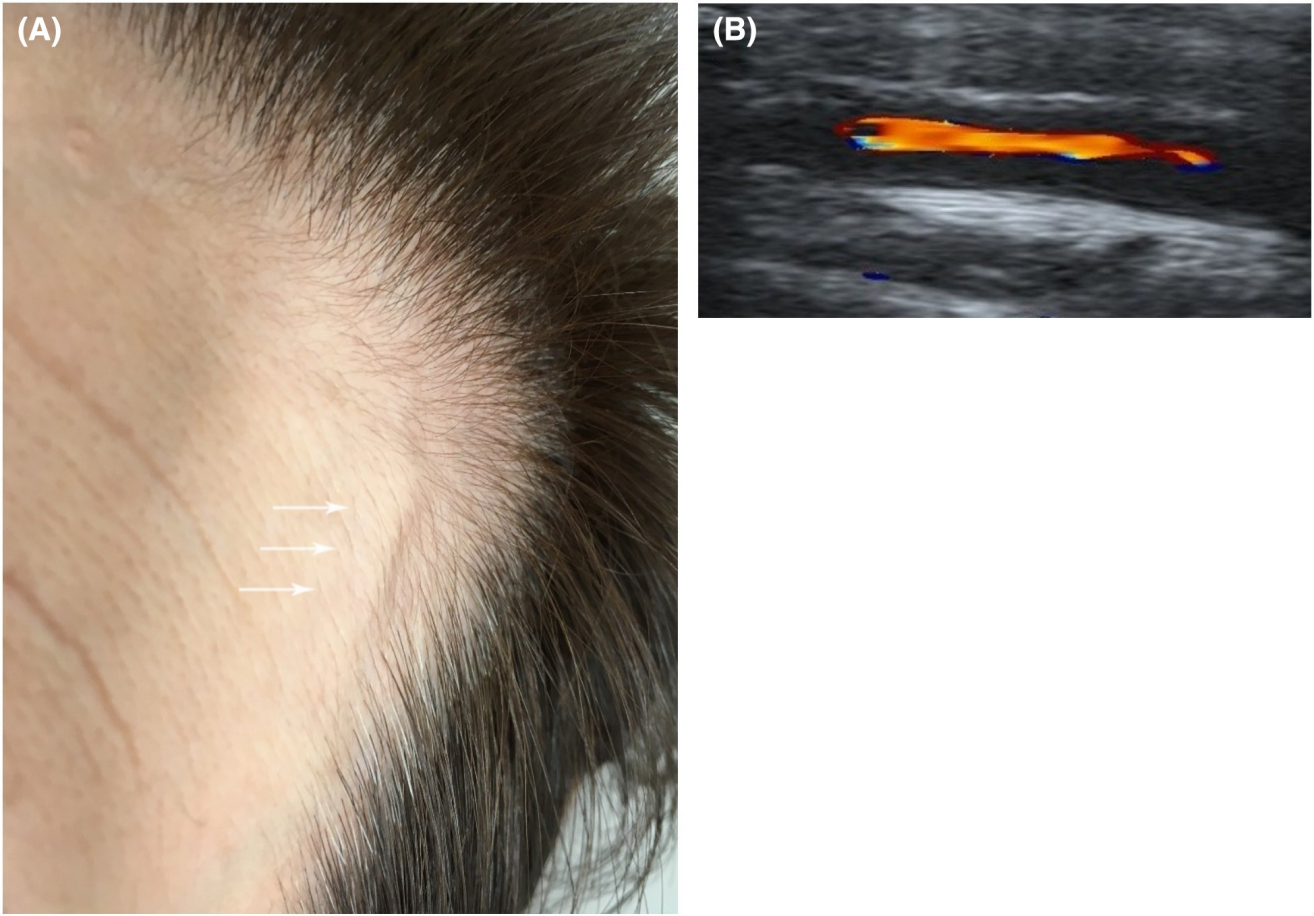
(A) Slightly enlarged left temporal artery (arrows). (B) Ultrasonography revealing wall thickening of the left temporal artery frontal branch.

Giant‐cell arteritis is a vasculitis that often affects medium‐ and large‐sized arteries and shows a variety of clinical manifestations (Table 1). Headache is present in over 80% of cases in a report.^2^ Our patient complained of toothache, but she had no problems with her teeth. Giant‐cell arteritis can also cause serious risks, such as blindness,^3^ and requires prompt diagnosis. Giant‐cell arteritis occurs less often in Japan, Korea, and other Asian countries than in Western countries; the reason for this discrepancy remains unclear. In Asian countries, GCA is one of the cause of fever of unknown origin. In primary care settings, the possibility of GCA should be considered in elderly Asian patients suffering from fever and pain in the temple; ultrasonography should be performed to detect wall thickening in the temporal artery frontal branches. This easy method is very useful for the diagnosis of this emergent disease.

**TABLE 1:** Clinical findings in 100 patients with giant‐cell arteritis (Modified quotation from Ref. 4).

Finding	Number
Gender (female/male)	69/31
Onset (gradual/sudden)	64/36
Weight loss or anorexia	50
Malaise, fatigue, or weakness	40
Fever	42
Polymyalgia rheumatica	39
Other musculoskeletal pains	30
Synovitis	15
Sore throat	9
Symptoms related to arteries	83
Headache	68
Visual symptoms	
Transient	16
Fixed	14
Jaw claudication	45
Swallowing claudication or dysphagia	8
Tongue claudication	6
Limb claudication	4
Signs related to arteries	66
Artery tenderness	27
Decreased temporal artery pulsations	46
Erythematous, nodular, or swollen scalp arteries	23
Large artery bruits	21
Decreased large artery pulses	7
Visual loss	14
Ophthalmoscopic abnormalities	18
Extraocular muscle weakness	2
Raynaud phenomenon	3
Central nervous system abnormalities	15

## FUNDING INFORMATION

None.

## CONFLICT OF INTEREST STATEMENT

No potential conflict of interest relevant to this article was reported.

## ETHICS APPROVAL STATEMENT

None.

## PATIENT CONSENT STATEMENT

We obtained informed consent from the patient for this case report.

## CLINICAL TRIAL REGISTRATION

None.
